# A comparative study of a rabbit spinal tuberculosis model constructed by local direct infection via the posterior lateral approach

**DOI:** 10.1038/s41598-022-16624-2

**Published:** 2022-07-27

**Authors:** Xuefeng Yue, Xi Zhu, Longyun Wu, Jiandang Shi

**Affiliations:** 1 Department of Orthopedics, The First People’s Hospital of Yinchuan, Liqun Street, Xingqing District, Yinchuan, 750001 Ningxia People’s Republic of China; 2 Department of Orthopedics, The Second Affiliated Hospital of Ningxia Medical University, Yinchuan, 750001 China; 3 Department of Spine Surgery, General Hospital of Ningxia Medical University, 804 Shengli Street, Xingqing District, Yinchuan, 750003 Ningxia People’s Republic of China

**Keywords:** Infection, Infectious diseases

## Abstract

The present study aims to establish a method of constructing a New Zealand rabbit spinal tuberculosis model by direct local infusion of *M. tuberculosis* H37Rv strain into the intervertebral disc space through the posterior lateral approach. Sixty-six New Zealand rabbits were pretreated with complete Freund's adjuvant and randomly divided into 4 group: the posterolateral approach model group (Group A, 25), ventral transverse process approach model group (Group B, 25), control group (Group C, 10), and blank group (Group D, 6). In Groups A and B, the bone holes were filled with gelatin sponge after drilling, and the local area was directly infused with 0.1 ml of *M. tuberculosis* H37Rv strain suspension. In Group C, the gelatin sponge was filled through the posterolateral approach and the local area was infused with 0.1 ml of normal saline suspension. In Group D, No specific treatment was performed. The general conditions of the experimental rabbits in each group were compared to those of a control group; the degree of vertebral body exposure, incision length, and complications of the two methods were compared; and the tuberculosis models were evaluated by imaging, histopathology, and bacterial culture. In Group A, the lateral side of the vertebral body was well exposed, the damage was mild, and no peritoneal rupture or gastrointestinal complications were observed. In Group B, the ventral side of the vertebral body and the intervertebral disc were exposed, and abdominal complications were more likely to occur. The survival rates of the experimental rabbits at 8 weeks after surgery were 92.0% in Group A, 88.00% in Group B, 90.0% in Group C, and 100% in Group D. MRI examinations showed that in Group A, the positive rate of radiographic bone findings was 86.9% at 4 weeks after surgery and 100% at 8 weeks after surgery; in Group B, the positive rate of radiographic bone findings was 78.2% at 4 weeks after surgery and 95.4% at 8 weeks after surgery. There was no significant difference between Groups A and B in the radiographic bone findings rate detected by the same imaging method at the same time point (*P* > 0.05). Eight weeks after surgery, bone destruction, paravertebral abscess, and caseous necrosis occurred in the vertebral bodies of surviving rabbits in Groups A and B. The BacT/ALERT 3D rapid culture system was used to culture the pus in the lesion, and the results showed that the positive rate of tuberculosis was 52.17% in Group A and 54.54% in Group B, and the difference was not statistically significant (*P* > 0.05). After pretreatment with complete Freund's adjuvant, direct infusion of the H37Rv strain of *M. tuberculosis* into the intervertebral disc space of New Zealand rabbits via the posterolateral approach and the ventral transverse process approach can successfully establish rabbit spinal tuberculosis models.

## Introduction

Spinal tuberculosis is still a major problem and an important public health issue that seriously endangers the health and quality of life of affected patients^1–5^. Although, animal models cannot completely stimulate the occurrence and development of human tuberculosis (TB), they play an important role in basic research on pathogenesis, new drug screening, vaccine development, and for a better nderstanding of host–pathogen relationships^6^. Various animals, including mice, guinea pigs, rabbits, and monkeys, have been used to model TB infection using pure cultures of *M. tuberculosis*^7^. Due to the complex anatomical structure of small bones, the spine and the intractability, inertia, and conditionality of *M. tuberculosis*, it is relatively difficult to establish animal models of spine tuberculosis. Rabbits may be an ideal animal model for spinal tuberculosis. because it is simple to perform basic surgical procedures on their spines , and their considerable susceptibility to *M. tuberculosis*. Geng Guangqi et al. ^8^ established an ideal spinal tuberculosis animal model by direct infusion of the *M. tuberculosis* H37Rv strain into the intervertebral disc space of New Zealand rabbits through the ventral transverse process approach and obtained a high success rate, but the surgical exposure is complicated and experimental animals face many complications. An improvement of the method was prompted and the New Zealand rabbit spinal tuberculosis model was constructed using the posterolateral approach. In this study, we selected New Zealand rabbits sensitized with adjuvants, infused the tuberculosis H37Rv strain directly into the intervertebral disc space via two different approaches, and successfully established rabbit spinal tuberculosis models.

## Materials and methods

### Ethical statement

In the current study, we handled the animals consistently in accordance with the ARRIVE guidelines. This study was approved by the Ethics Committee of Ningxia Medical University. All experiments were carried out in accordance with the authorised experimental protocols and the Guide for Care. Throughout the experiments, the rabbits were maintained under controlled conditions. The relative humidity was controlled at 60% ± 80%, a 12/12 h dark/light cycle was maintained, and the ambient temperature was 21 °C ± 25 °C. The cages and the drinking water and food items required for the experimental animals were sterilized beforehand, and the experimental animals were allowed to eat and drink freely. Before each surgery, the rabbits were fasted overnight and then anaesthetized.

### Pretreatment and grouping of experimental animals

Sixty-eight healthy New Zealand rabbits (provided by the Experimental Animal Center of Ningxia Medical University) that had a negative purified protein derivative test (PPD skin test) for *M. tuberculosis* were selected. The body weights ranged from 2.5 to 2.8 kg. These New Zealand rabbits were injected with 0.1 ml of complete Freund's adjuvant intracutaneously on the back of the neck. Model establishment was carried out after one month of sensitization. Two of the experimental rabbits were excluded due to ulceration of the neck skin. The rest of the experimental rabbits that had intact skin and local induration (diameter: 5–20 mm) were included in this study. These rabbits were divided into 4 groups: the posterolateral approach model group (Group A, 25), ventral transverse process approach model group (Group B, 25), control group (Group C, 10), and blank group (Group D, 6).

### Establishment of a rabbit spinal tuberculosis model by direct local infection

#### Bacterial strains

The *M. tuberculosis* H37Rv strain was cultured and expanded in Löwenstein-Jensen (LJ) medium for 3 weeks to enter the logarithmic growth phase. A sterile "L-shaped" glass rod was used to collect the cauliflower-like colonies and a glass grinder was used to grind and mix them in a sterile 0.9% sodium chloride solution. The bacterial suspension was filtered through a 5 µm sterile filter, and the bacterial density was adjusted to 1 × 107 colony forming units (CFU)/ml. Counting was carried out on LJ medium and the suspension was stored at − 80 °C until further use.

#### Direct local infection by bacterial inoculation

Rabbits distributed in Groups A, B, and C were treated according to the following methods, while no treatment was applied to the rabbits in Group D (blank group).

**Figure 1:**
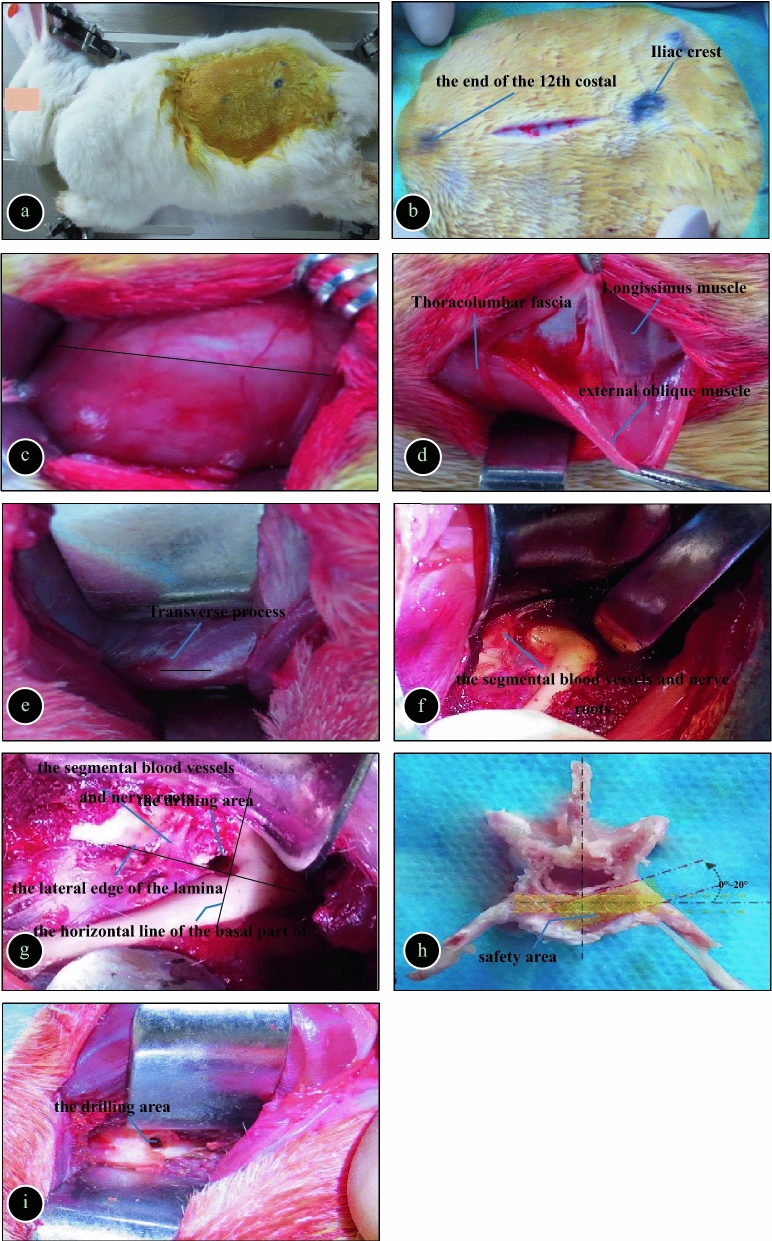
(**a**) Surgical position; (**b**) Surgical incision; (**c**) The thoracolumbar fascia was cut along the dotted line (red and white); (**d**) The intramuscular space between the longissimus and obliques was dissected bluntly; (**e**) The apex of the transverse process was exposed; (**f**) The vertebral body and the lateral side of the intervertebral disc were exposed, and segmental blood vessels and exiting nerve roots could be seen; (**g**) The drilling site of the posterolateral approach; (**h**) The drilling angle and safety zone of the posterolateral approach; (**i**) The drilling site of the ventral transverse process approach.

1. *Anaesthesia* 3% sodium pentobarbital (30 mg/kg) was slowly injected via the ear vein, and supplemental local infiltration anaesthesia was carried out with 5–10 mL of 2% lidocaine injection during surgery.
2. *Position* Skin preparation was carried out on the left side of the waist and abdomen (15 cm × 15 cm), and the rabbit was placed in the right lateral decubitus position on the operating table without fixation.
3. *Incision* The surgical site was prepared routinely. A 6 cm longitudinal incision was made along the end of the left 12th rib down to the iliac crest. The midpoint of the incision was at the L4-5 spinal level (the junction between the line connecting the highest points of the bilateral iliac crests and the line connecting the L5 and L6 spinous processes).
4. *Exposure* The skin was cut open, the subcutaneous fascia was dissected layer by layer, the thoracolumbar fascia (TLF) was cut along the space between the longissimus muscle and the external abdominal muscle, and the intermuscular space was dissected using a blunt dissection technique to expose the transverse process as shown in Fig. 1a–d.
5. Direct local infection in each group for the establishment of a rabbit spinal tuberculosis model.
  (5.1) *Posterolateral approach model group (Group A)* the attachment of the longissimus muscle extended from the transverse process was incised using a sharp scalpel blade, and the longissimus was dissected along the dorsal side of the transverse process to expose the lateral side of the intervertebral disc, where the segmental blood vessel and exiting nerve root could be seen. The lateral margin of the lamina and the upper margin of the transverse process were taken as the boundary, and the upper inner quadrant was drilled. The drilling direction was inclined to the dorsal side at an angle of 0°–20°. A rongeur was used to remove the cortical bone in the drilling area to prevent slipping, and then a bone hole with a diameter of 2 mm and a depth of approximately 1 cm was made by drilling. The bone hole was filled with a gelatin sponge after haemostasis, 0.1 ml of 5.0 mg/ml tuberculosis suspension was infused with a syringe into the absorbable gelatin sponge, and the intervertebral disc was punctured with the puncture needle carrying bacteria. Finally, the bone hole was sealed with surgical bone wax (Fig. 1e–h).
  (5.2) *Ventral transverse process approach model group (Group B)* A nerve stripper was used to dissect the ventral side of the transverse process longitudinally, and the spine was turned with appropriate force to expose the vertebral body and the front of the intervertebral disc. A bone hole was drilled from the left-rear to the right-front at the upper part of the L5 vertebral body approximately 0.5 cm away from the L4-5 intervertebral disc. The bone hole was drilled at a 30° angle to the distal end of the vertebral body (the depth was approximately 0.5 cm, and the diameter was approximately 0.3 cm). The bone hole was filled with a gelatin sponge after haemostasis, and 0.1 ml of 5.0 mg/ml tuberculosis suspension was infused with a syringe into the absorbable gelatin sponge. Finally, the bone hole was sealed with bone wax (Fig. 1e, i).
  (5.3) *Control group (Group C)* A bone hole was drilled via the posterolateral approach and filled with a gelatin sponge. Then, 0.1 ml of normal saline was infused into the gelatin sponge.
  (5.4) *Blank group (Group D)* No specific treatment was performed.
6. The incision wound was closed with silk interrupted sutures, which were covered with sterile gauze and fixed.

### Observation indicators and evaluation standards

#### Observation indicators

1. The following indicators were observed during surgery and immediately after surgery in Groups A and B: exposure time, incision length, and early postoperative complications.
2. Observation of postoperative conditions: the activity, mental state, and eating behaviour of the experimental rabbits, whether the wound has redness, swelling, infection, or abscess, and whether there is paraplegia.
3. Imaging observations: the experimental rabbits of each group successfully underwent imaging examinations (plain X-ray, CT, MRI) on the first day and at 4–8 weeks after model establishment to observe the destruction of the intervertebral discs and vertebral bodies; and the formation of sequestrum and the abscess.
4. Histopathological observation: the experimental rabbits were sacrificed at 8 weeks after the surgery, the changes in the infected lumbar vertebral body and intervertebral disc; and the paravertebral abscess in each group were observed aseptically, and histopathological examination using haematoxylin and eosin (HE) staining was performed routinely.
5. When *M. tuberculosis* was cultured for 8 weeks, 0.5 g of pus in the destruction area was harvested aseptically and placed in a culture flask after pretreatment. The liquid culture medium containing the specimen was incubated in a BacT/ALERT 3D incubator, at a constant temperature of 37 °C. During that period, the results shown by the incubator were observed, and the specimen was finally cultured for 6 weeks. When *M. tuberculosis* grew, it metabolized and produced CO^2^, which caused pH changes and made the indicator change colour. The results were detected and recorded automatically and continuously by the device.

### Statistical analysis

SPSS 17.0 software was used for statistical analysis. The evaluation indicators obtained were expressed as the mean ± standard deviation. The *t test* was used to compare the means of two groups, the *ANOVA* was used to compare the means of three groups. and the count data were compared by Fisher's exact test. The p-values were corrected for multiple testing using FDR correction. False Discovery Rate (FDR) *q-values* less than 0.05 were considered significant.

## Results

### Intraoperative observation

In Group A, the lateral side of the lumbar vertebrae was better exposed, the exposure time was shorter (5.98 ± 0.58 min), the operation was simple, the injury was mild, and there were no peritoneal ruptures or common complications of the digestive tract (Table 1).

**Table 1:** Early postoperative observation and imaging evaluation of experimental rabbits in each group.

Projects	Groups				*P* (compare ^①^and^②^)
	The A group ^①^	The B group ^②^	The C group	The D group	
Exposure time (min)	5.98 ± 0.58	7.72 ± 1.07	5.83 ± 0.73	–	0.000
**Operative incision (cm)**	6.46 ± 0.49	6.84 ± 0.37	6.07 ± 0.48	–	0.000
Injury of vertebral artery branch in operation	5	1	1	–	0.198
Injury of spinal cord in operation	0	1	0	0	1.000
Peritoneum rupture	0	4	0	0	0.110
Digestive tract symptoms such as no defecation and no eating after operation	0	7	0	0	0.004
Paraplegia (8 weeks after operation)	1	2	0	0	0.968
**Number of rabbits with positive spinal tuberculosis evaluated by imaging (n/N)**					
4 W: X-ray (narrowing intervertebral space, bone destruction, etc.)	12/23(52.1)	11/23(47.8)	0	0	0.768
8 W: X-ray (narrowing intervertebral space, bone destruction, etc.)	14/23(60.8)	13/22(59.0)	0	0	0.903
4 W: CT (dead bone, calcification, paravertebral abscess, etc.)	19/23(82.6)	17/23(73.9)	0	0	0.721
8 W: CT (dead bone, calcification, paravertebral abscess, etc.)	22/23(95.6)	21/22(95.4)	0	0	1.000
4 W: MRI (dead bone, calcification, paravertebral abscess, etc.)	20/23(86.9)	18/23(78.2)	0	0	0.697
8 W: MRI (dead bone, calcification, paravertebral abscess, etc.)	23/23(100)	21/22(95.4)	0	0	0.489

In Group B, the vertebral body and the ventral side of the intervertebral disc were fully exposed, but the exposure time was relatively long (7.72 ± 1.07 min), and digestive tract symptoms were more likely to occur (Table 1).

### Postoperative observation

Except for one experimental rabbit in Group C which died on the same day after surgery and one rabbit in the Group B which suffered spinal cord injury during surgery, the rest of the rabbits recovered smoothly from anaesthesia. In Group A, the food and water intake of rabbits became normal during the early postoperative recovery, and the incisions healed well; one experimental rabbit died 3 weeks after surgery, the autopsy revealed a large amount of pus in the thoracic cavity, and the acid-fast staining of the pus was positive; another rabbit did not wake up from anaesthesia during imaging examination at 4 weeks after surgery; one rabbit developed paraplegia, and imaging examination showed that the abscess compressed the spinal cord and dural sac. In Group B, the food and water intake of 7 experimental rabbits decreased during the early postoperative recovery; 2 rabbits (including paraplegic rabbits) showed intermittent symptoms such as nondefecation, weight loss, anorexia, poor mental state, and decreased activity levels and died at 7 days and 23 days after surgery; the remaining 5 rabbits gradually regained normal appetite; one rabbit had poor incision healing, sinus formation, and formation of caseous necrosis, which was sacrificed to prevent the spread of disease; the later autopsy showed a large amount of pus in the abdominal cavity, and the acid-fast staining of the pus was positive; another rabbit developed paraplegia at 6 weeks after surgery, and imaging examination showed that the abscess compressed the spinal cord and dural sac. In Groups C and D, the surviving rabbits had normal food and water intake during early postoperative recovery, and the incisions healed well. Comparison of the survival rates of Groups A, B and C showed that there were no statistically significant differences (*P* > 0.05) (Table 2).

**Table 2:** Survival status of experimental rabbits in each group after modelling establishment.

Groups	Survival conditions of experimental rabbits at each time point			
	Premodelling quantity (1 month after immune pretreatment)	The 4th W after modelling	The 8th W after modelling	The survival rate at the 8th W
The A group	25	23	23	0.92^①^
The B group	25	23	22	0.88^②^
The C group	10	9	9	0.90
The D group	6	6	6	1.00

Compare ① and ②, *P* > 0.05.

### Imaging observation

**Figure 2:**
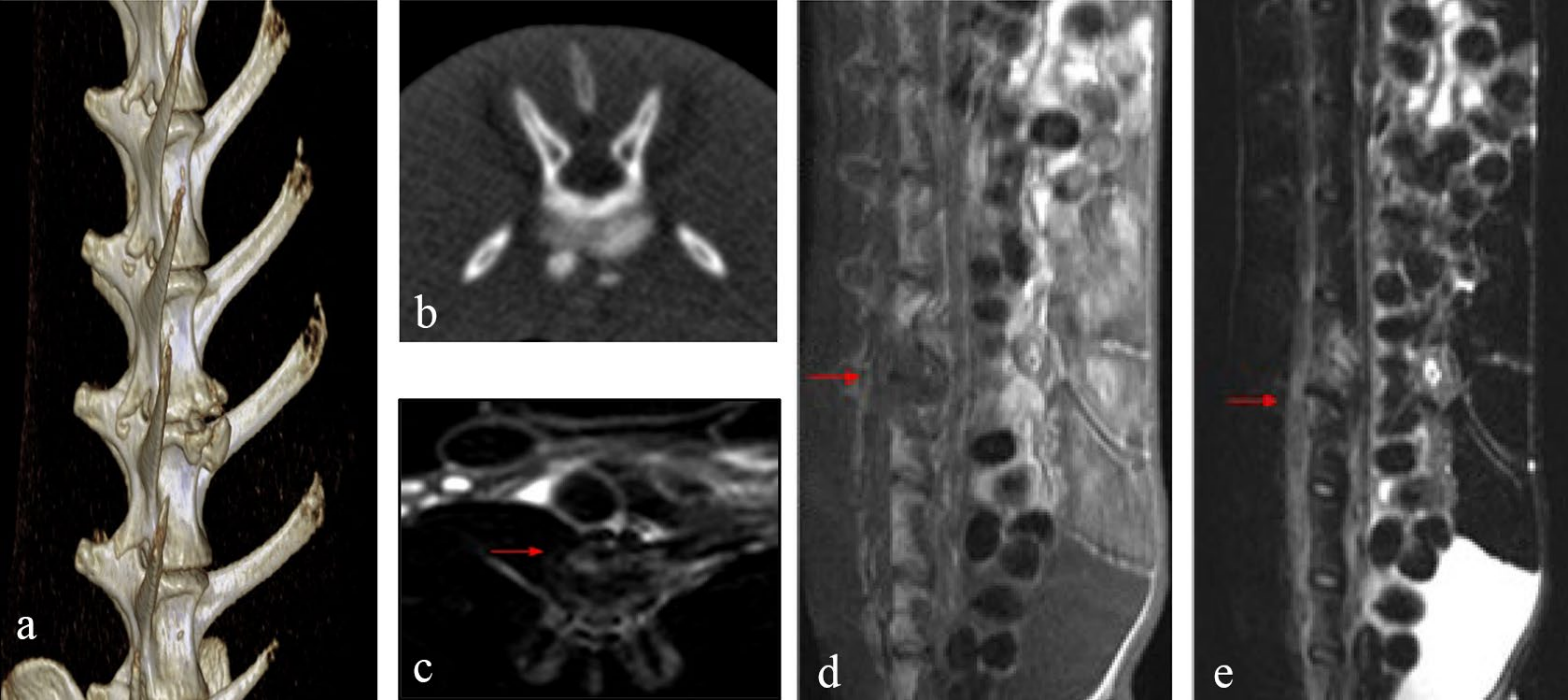
A New Zealand rabbit model of spinal tuberculosis (imaging data): (**a**) CT reconstruction showed narrowing of the intervertebral disc space, destruction of adjacent vertebral bodies, marginal and worm-like destruction of the affected vertebral body; (**b**) CT scan showed vertebral bone destruction; (**c**) Swelling of the psoas major muscle and paravertebral abscess; (**d**–**e)** Long low-intensity signals on T1WI and mixed high-intensity signals on T2WI in the sagittal view.

In Groups A and B, the imaging observations showed dynamic changes on the next day, at 4 weeks and 8 weeks after surgery, which manifested as progressive radiographic bone findings, and the extents of tuberculosis lesions on a single vertebra were more consistent (Fig. 2). X-ray findings were mainly characterised by narrowing of intervertebral disc space, blurred articular surface, and spinal deformity; CT scan findings were intervertebral disc space narrowing, sequestrum, calcification, bone destruction, paravertebral abscess, and compression of dura mater and spinal cord; MRI showed long low-intensity signals on T1WI and mixed high-intensity signals on T2WI in the sagittal view at 4 weeks, and mixed long T1 signals and long T2 signals in the sagittal view at 8 weeks, while it showed psoas abscess and intervertebral disc space narrowing in some rabbits. The positive rate of MRI for radiographic bone findings of spinal tuberculosis was higher than that of CT and X-ray imaging. The MRI examinations showed that in Group A, the positive rate of radiographic bone findings was 86.9% at 4 weeks after surgery, while it was 100% at 8 weeks after surgery; in Group B, the positive rate of radiographic bone findings at 4 weeks after surgery was 78.2%, while it was 95.4% at 8 weeks after surgery. There was no statistically significant difference between Groups A and B in the positive rate of radiographic bone findings at the same time point (*P* > 0.05). The experimental rabbits in the control group (Group C) had no changes in the intervertebral disc space and no signs of vertebral body destruction on the next day, at 4 weeks and 8 weeks after surgery. No significant abnormalities were detected by the imaging examinations in Group D. Detailed data are presented in Table 1.

### Histopathological observation

**Figure 3:**
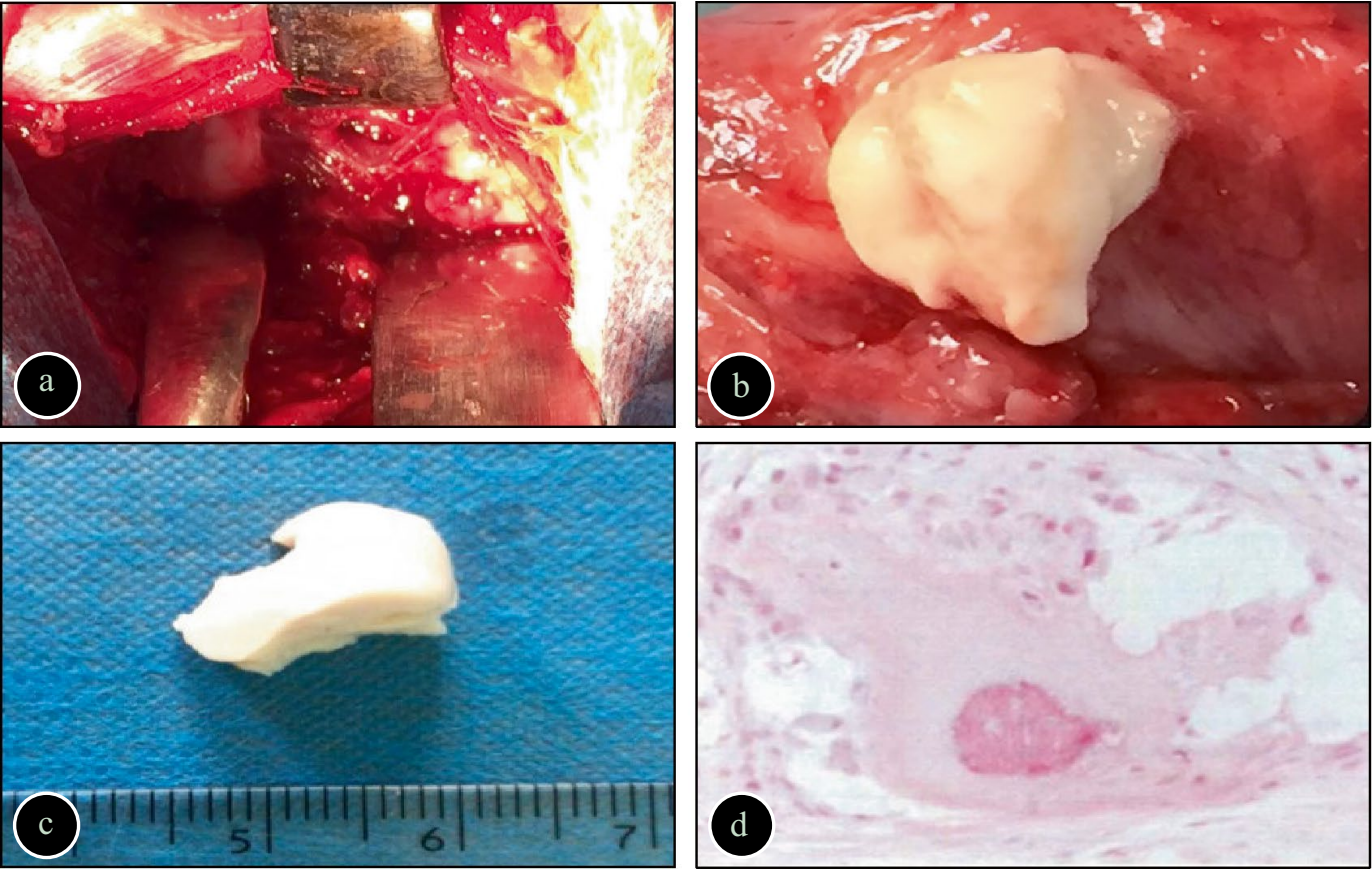
(**a**) A spinal tuberculosis lesion (vertebral bone destruction and paravertebral abscesses could be seen); (**b**) Abscess formation; (**c**) Caseous necrotic substance; (**d**) Bone depressions within the affected vertebrae, and fibrous tissue around the lesion.

The experimental rabbits in Groups A and B were dissected under anaesthesia 8 weeks after surgery, which showed vertebral bone destruction and paravertebral abscess formation (Fig. 3). In Group C, the drilled bone hole was closed, and there were no obvious signs of bone destruction in the vertebral body or other adjacent vertebrae. The lumbar spine tuberculosis lesions of rabbits in Groups A and B were mainly osteolytic bone destruction. There were scattered mononuclear macrophages, trabecular bone fracture, erosion, resorption and, disappearance in the lesion. Tuberculous granulomas and scattered macrophages could be seen between trabecular bones, and the lesion contained scattered osteoclasts infiltrated by inflammatory cells. The tissue staining of the caseous, necrotic substance showed consistent unstructured necrotic masses, which were presented as red-stained and unstructured granular materials. Microscopic examination of the normal vertebral cancellous bone showed that the structure of bone trabeculae was regular, the osteoblasts were distributed evenly, and there was no sequestrum and or changes in epithelial cells.

### *M. tuberculosis* culture

At 8 weeks after surgery, 0.5 g of pus was taken aseptically from the destroyed area and placed in a culture bottle after pretreatment. The liquid culture medium containing the specimen was placed in the BacT/ALERT3D incubator, and incubated at a constant temperature of 37 °C. During this period, the results were observed, and the specimen was finally cultured until the 6th week. After incubation, the percentages of positive cultures in Group A and Group B were 52.17% and 54.54, respectively. Bone damage findings matched animals that were positive for growth in cultures.

## Discussion

Spinal tuberculosis is usually a result of haematogenous spread of *M. tuberculosis* into the dense vasculature of cancellous bone of the vertebral bodies. When the body's immune response is reduced or other unfavourable factors exist, latent *M. tuberculosis* rapidly multiplies, and a series of complex pathophysiological changes occur, which eventually lead to bone destruction of the vertebral body. It is still the goal of many scholars to explore the key factors such as the route of infection and the immunity of infected animals, and to establish a reasonable and reliable animal model to reproduce this complicated pathological change.

### Selection of laboratory animals

Currently, the main animal models commonly used for TB research are guinea pigs, mice, rabbits and non-primate animals, each with different advantages and disadvantages. The guinea pig is very sensitive to *M. tuberculosis* and has been widely used for tuberculosis research. Mice have a clear genetic background, it is easy to access reagents for them, they are inexpensive, and there exists relatively sophisticated targeted gene knockout technology; therefore, mice are commonly used as models for studying mutations in *M. tuberculosis*, efficacy assessment, and vaccine screening. However, guinea pigs and mice have small vertebrae and are not suitable for surgery such as bone grafting, and they are not suitable as animal models for spinal tuberculosis. The biological and behavioural characteristics of nonhuman primates are similar to those of humans. They play an important role in basic and preclinical research related to human diseases and are the most ideal experimental animals for studying tuberculosis. However, their use is somewhat limited due to the high cost and difficulty of raising them in the laboratory. Because the rabbit lumbar anatomy and sensitivity to tuberculosis are similar to those of humans, its genetic information is complete and reproducible, and it is more economical and easy to be obtained, so rabbits are often used to replicate many unique stages of spinal tuberculosis^9,10^.

### Choice of local infection route of the vertebral body

The establishment of the bone tuberculosis model by direct local infection is considered to be a reliable method, which has the advantages of clear lesion location and a short period of model establishment. There are many methods for puncturing or exposing the intervertebral disc. Bierry G et al.; succeeded in directly injecting *Staphylococcus aureus* into the intervertebral disc percutaneously under fluoroscopy procedures. However, auxiliary fluoroscopy is needed during the procedure, which increases the operator's risk of exposure to radiation^11^. The posterior approach through the longissimus and multifidus muscles can expose the posterior part of the vertebral body and the transverse process. However, this approach may cause massive internal bleeding and serious injury, and the required prone position increases abdominal pressure, which affects the surgical procedure and anaesthesia management^12–14^. Geng Guangqi et al.; used the ventral transverse process approach to expose the vertebral body and intervertebral disc, and they successfully established a model establishment by drilling a hole in the upper endplate of the vertebral body and, filling it with gelfoam sponge infused with H37Rv standard *M. tuberculosis* suspension. The New Zealand rabbit model of spinal tuberculosis using the same or similar methods has been used in basic research related to spinal tuberculosis with satisfactory results^15,16^. In previous in vivo experiments using this method, the results showed that repeated surgeries caused local adhesion, unclear anatomical structure boundaries, and a corresponding increase in the incidence of surgical complications such as peritoneal rupture. The improvement of the method was prompted and the New Zealand rabbit spinal tuberculosis model was constructed using the posterolateral approach. In the present study, we set up the ventral transverse process approach group and found that this approach could better expose the vertebral body and the front of the intervertebral disc with less bleeding and a higher success rate in model establishment. However, because the transverse process is relatively long and extends to the anterior and upper sides in an arc, to increase the exposure of the vertebral body, the spine needs to be turned in place, which may cause the risk of transverse process fracture on the same side and rupture of the peritoneum. Moreover, digestive tract symptoms are more likely to occur under the influence of surgical factors, including application of the intestinal spatula. Model establishment via the posterolateral approach was performed on the dorsal side of the transverse process, retraction was easy, surgical exposure was satisfactory, and digestive tract complications were less likely to occur because the surgical field was located a certain distance from the abdominal organs. However, due to the small size of the rabbit vertebral body, the drilling area through the posterolateral approach is close to important organs, so it is necessary to fully grasp the anatomy of the rabbit spine before the operation, and multiple pretests before surgery and it is important to pay attention to the following: ① The operation should be performed under direct vision, and only the key anatomical structures should be exposed; ② Dissection and exposure should be carried out bluntly along with the intermuscular space, the manipulation should be gentle to avoid accidental injury to the segmental blood vessels, if it occurs, immediate haemostasis should be carried out. ③ The cortical bone in the drilling area needs to be removed in advance to prevent the drill bit from slipping into the spinal canal to damage the spinal cord. During the operation, the drilling must be slow and the drilling angle must be controlled to prevent drilling into the spinal canal.

### Adjustment of the immune status of experimental animals

In 1969, Hodgson et al.^17^ injected *M. tuberculosis* into the paravertebral tissues of rabbits and found that animal mortality was high due to the rapid spread of *M. tuberculosis*. Liu Xiaochen et al.^18^ drilled holes in the vertebral bodies of unsensitized New Zealand rabbits, inserted gelatin sponges containing *M. tuberculosis* into the bone holes, sealed them with bone wax, and established a spinal tuberculosis model that was more consistent with human diseases, but the success rate of the model established was only 68.1%. After tuberculosis bacteria enter the body, most of them are swallowed and killed by macrophages or cause a tuberculosis infection state without clinical symptoms. Therefore, the key before establishing a spinal tuberculosis model is to understand and adjust the immune status of experimental animals, so that the animal's immune status is at a reasonable level, which can cause disease without inducing the spread of tuberculosis throughout the body and resulting in animal death. Freund's adjuvants are currently the most commonly used adjuvants in animal experiments and can nonspecifically change or enhance the body's specific immune response to antigens. In this study complete Freund's adjuvant was used for pretreatment, and it was confirmed that it can prolong the action time of the antigen so that the antigen stays in the infected area, is slowly and persistently released, and continues to stimulate and enhance immunogenicity and reduce the haematogenous spread of *M. tuberculosis*. In addition, it further aggravates the pathophysiological progress of bone destruction and inflammatory exudation, which is more consistent with the characteristics of human bone tuberculosis. This method is simple, the number of bacteria is easy to control, the cycle of model establishment is short, the success rate and reproducibility are high, individual differences are small, and the disease severity is more consistent.

### Evaluation and screening of rabbit spinal tuberculosis model

An ideal animal model of spinal tuberculosis should have the following characteristics: ① The survival rate of experimental animals is high; ② The model can simulate the pathological process of human diseases, the lesion and bone destruction are consistent, and the reproducibility is high; ③ There is no tuberculosis in other parts including the and abdomen; ④ There is a single lesion involving only the anterior column of the vertebral body, the vertebral body is relatively stable, and there is no paraplegia.

Comprehensive evaluation methods based on imaging, pathology, microbiology examinations, etc. should be used to screen animal models of spinal tuberculosis. In this study, bone damage findings did not matched between the different imaging modalities. CT imaging is superior to X-ray imaging for demonstrating the presence of paraspinal abscesses. CT scan examination can clearly show sequestrum, calcification, and small hidden bone destruction in the lesion. Compared with X-rays and CT imaging, MRI is more sensitive for diagnosing spinal tuberculosis. MRI can show oedema and necrosis in the tuberculosis-infected vertebral body earlier. In the present study, typical adjacent vertebral body destruction, narrowing and disappearance of the intervertebral disc space, intervertebral fusion, and vertebral body destruction were observed 4 weeks after surgery. With the rapid proliferation of tuberculosis strains in the lesion, postoperative dynamic imaging showed signs of progressive bone destruction, but the lesions in the same group at the same time point were more consistent.

Pathological examination diagnoses diseases infected by tuberculosis, clarifies the range of the lesion, and helps us to better understand the development process and specific changes of the lesion. In this study, the rabbits were dissected 8 weeks after surgery, and typical tuberculosis lesions, such as typical paravertebral abscess, bone destruction, and caseous necrosis, were observed. Meanwhile, HE staining of the bone tissue showed osteolytic destruction, and HE staining of the caseous necrosis showed consistent and unstructured necrotic changes.

The bacterial culture of *M. tuberculosis* is an important indicator for evaluating animal models^19^. Approximately 8 weeks after surgery, the pus in the lesion was harvested aseptically and cultured. The positive rate of the culture of the posterolateral approach model was 52.17%; and that of the ventral transverse process approach model was 54.54%. In this study, except for the two rabbits who died of paraplegia caused by abscess-induced spinal cord compression and combined tuberculosis in other parts, the remaining experimental rabbits were confirmed to be ideal models with consistent lesions and bone destruction.

Rabbits in this study were pretreated with complete Freund's adjuvant, and the *M. tuberculosis* H37Rv strain was injected directly into the target area, which successfully established spinal tuberculosis animal models most similar to humans. In the present study, the economical New Zealand rabbits were selected as experimental animals; the posterolateral approach and the ventral transverse process approach were used for the injection of bacterial strains into the vertebral body, and each method had its advantages and disadvantages. Adjuvants used to adjust the immune status of the experimental animal could increase the success rate of model establishment, the procedure was simple and easy to perform, and it was cost effective. After screening and evaluation, the lesion and bone destruction of the model were consistent, and the repeatability and reliability were high. However, this study did not investigate blood inflammatory indicators in the animal model and rapid detection of tuberculosis-specific infections was not performed. Additional research will be needed to resolve these issues.

## Abbreviations

LJ: Lowenstein-Jensen
HE: Haematoxylin and eosin
PPD: Purified protein derivative test
CFU: Colony forming units
TLF: Thoracolumbar fascia
BJ: Bone and joint
